# LncRNA OR2A1‐AS1 index predicts survival in germinal center‐like diffuse large B‐cell lymphoma

**DOI:** 10.1002/jcla.24680

**Published:** 2022-09-04

**Authors:** Shuting Ye, Weiwei Ying, Yi Lin, Zhengjun Hou, Meiyun Su

**Affiliations:** ^1^ Department of Hematology The First People's Hospital of Wenling Wenling China; ^2^ Department of Pathology The First People's Hospital of Wenling Wenling China; ^3^ Department of Traditional Chinese Medicine The First People's Hospital of Wenling Wenling China

**Keywords:** DLBCL, lncRNA, OR2A1‐AS1

## Abstract

**Background:**

Diffuse large B‐cell lymphoma (DLBCL) is a highly aggressive form of non‐Hodgkin lymphoma. Long noncoding RNA (lncRNA) has been evaluated as prognostic markers in various carcinomas. However, the prognostic value of the lncRNA index in DLBCL has not been fully understood. Hence, this study aimed to identify the prognostic value of lncRNA olfactory receptor family 2 subfamily A member 1‐antisense RNA 1 (OR2A1‐AS1) in DLBCL.

**Methods:**

The Gene Expression Omnibus (GEO) database was used to obtain the GSE97336 dataset comprising lncRNA expression profiles. Quantitative reverse transcription polymerase chain reaction (QRT‐PCR) was conducted to evaluate the expression of OR2A1‐AS1 in 98 cases of DLBCL.

**Results:**

OR2A1‐AS1 expression was considerably reduced in DLBCL patients, reduced OR2A1‐AS1 expression was linked to a shorter overall survival (OS) and progression‐free survival (PFS) in DLBCL patients, especially those with the germinal center B‐cell‐like subtype (GCB). Multivariate analysis (MVA) revealed that the OR2A1‐AS1 index had prognostic significance. Patients with low OR2A1‐AS1 expression have a poor prognosis.

**Conclusions:**

OR2A1‐AS may represent an effective predictor of patients' outcomes with DLBCL.

## Graphical abstract

In the study, the OR2A1‐AS index was found to be an effective predictor of patients' outcomes with DLBCL, particularly in the GCB‐DLBCL group. OR2A1‐AS was found to be a significant predictor of PFS in DLBCL in MVA. Targeting OR2A1‐AS treatments could be a promising method to improve patient outcomes in the age of precision medicine.

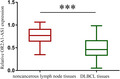

## 1 INTRODUCTION

Diffuse large B‐cell lymphoma (DLBCL) is a highly aggressive form of non‐Hodgkin lymphoma (NHL)^1^, ^2^, ^3^ and is considered to be diverse in terms of biological, clinical, and pathological features.^3^, ^4^, ^5^ The curative and survival rate was considerably enhanced in DLBCL patients by adding rituximab to CHOP (cyclophosphamide, rituximab, vincristine, doxorubicin, and prednisone) chemotherapy.^6^, ^7^ However, poorer survival was also observed in some DLBCL patients under standard R‐CHOP therapy.^8^

In recent decades, it has been revealed that long‐noncoding RNAs (lncRNAs) significantly contribute to regulating several biological events, such as proliferation, differentiation, and tumor suppression.^9^, ^10^ The dysregulated LncRNAs considerably contribute to cancer progression.^11^, ^12^ The role of lncRNA was recently identified in DLBCL.^13^ It has been demonstrated that LncRNA metastasis‐associated lung adenocarcinoma transcript 1 (MALAT1) sponged miR‐195 to improve tumor progression and immune escape of DLBCL.^14^ Through the miR‐34b‐5p‐GLI1 cascade, MYC‐regulated nuclear paraspeckle assembly transcript 1 (NEAT1) increased DLBCL proliferation.^15^ Another reported study has been revealed that the expression of lncRNA, i.e.*,* PEG10 was elevated in DLBCL relative to the normal tissues,^16^ which could provide a novel therapeutic target for DLBCL. However, there is a lack of clarity regarding the prognostic role of lncRNAs in DLBCL. Furthermore, the predictive value of some prognostic factors varied after the introduction of rituximab, a CD20 monoclonal antibody, and highlighting the need to reevaluate the prognostic value of predictive factors.^17^, ^18^

Microarray analysis was used to identify lncRNAs, and OR2A1‐AS1 were mostly decreased in DLBCL cell. Thus, the goal of this study was to determine the best OR2A1‐AS1 index prognosis cutoff value in DLBCL patients, to validate OR2A1‐AS1's specific prognostic value, and to research relationship with the cell of origin categorization (COOC). We also looked into whether the OR2A1‐AS1 index plays an effective role in DLBCL patients' prognosis.

## 2 MATERIALS AND METHODS

### 2.1 Patient selection

The current study included 98 patients with diagnosed DLBCL from the First People's Hospital of Wenling. We applied the Lymph2Cx assay using a NanoString gene expression platform on pretreatment tissues obtained from 98 patients with DLBCL. Correspondingly, 98 noncancerous lymph node tissues were collected. The research project was authorized by the Institute Research Ethics Committee of First People's Hospital of Wenling after all patients gave their informed consent in compliance with the Declaration of Helsinki's standards. All patients agreed to donate their samples to this research and completed a consent form. RCHOP‐like therapy was administered to all patients.

### 2.2 Bioinformatics analysis

The Gene Expression Omnibus (GEO) database (http://www.ncbi.nlm.nih.gov/geo/) was used to obtain the GSE97336 dataset comprising lncRNA expression profiles. Microarray analysis was used to identify lncRNAs that were differentially expressed in CD19‐positive B cells from OCI‐ly1 and OCI‐ly19 cells. The microarray type used in the investigation was GEO2R, which was obtained from GEO. ASHGA5P019110 (OR2A1‐AS1) were mostly decreased in DLBCL cell compared with CD19‐positive B cell.

### 2.3 qRT‐PCR evaluations

The OR2A1‐AS1 expression levels of target genes were determined using a qRT‐PCR. TRIzol™ (Invitrogen) was used for extracting total RNA from tissue samples, and 1 μg RNA was utilized for cDNA synthesis using the Reverse Transcription Kit (Takara). The ABI ViiATM7Dx Real‐Time PCR System (Life Technologies) was used to run the qRT‐PCR using the SYBR Green Realtime PCR Master Mix Kit (Toyobo). Relative expression was normalized using GAPDH and expressed using the 2^−ΔΔCT^ method.^19^ The underlined primers were used for the qRT‐PCR: OR2A1‐AS1, forward 5'‐ACGTGCACAGACAGCTAAGA‐3' and reverse 5'‐ATCATCCACGGGAGTGACGA‐3'; GAPDH (internal reference), forward 5'‐TGACTTCAACAGCGACACCCA‐3' and reverse 5'‐CACCCTGTTGCTGTAGCCAAA‐3'. Primers were designed using Primer3Plus (http://www.primer3plus.com/cgi‐bin/dev/primer3plus.cgi).

### 2.4 Statistical analysis

The primary endpoints of the current study were OS and PFS. GraphPad Prism 7 (La Jolla) was used for statistical evaluations. The Kaplan–Meier method was used to plot the survival curves, and the log‐rank test was carried out to compare them. A two‐tailed log‐rank test was conducted to identify variations, and a statistically considerable variation was defined as *p* < 0.05.

## 3 RESULTS

### 3.1 Profiling of lncRNAs from the DLBCL cell

**FIGURE 1:**
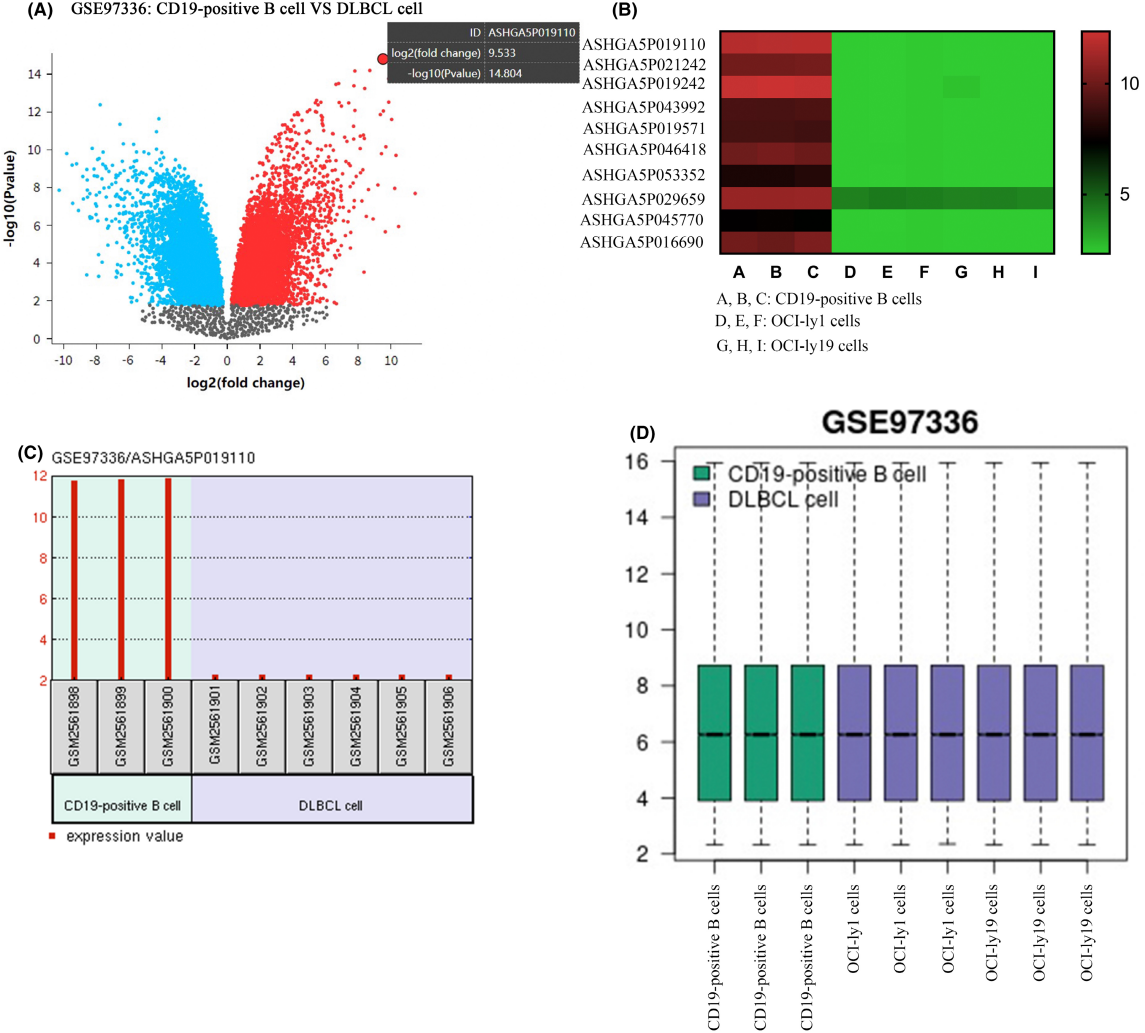
Profiling of lncRNAs from the DLBCL cell. (A) The volcano plot showed ‐log10 P‐values (Y‐axes) and log2 fold changes (X‐axes). Among them, the upregulated and downregulated genes have been represented by the red and blue colors, accordingly, while the black color represents no considerable variation in genes. (B) In the cluster graph of the samples, red, black, and green colors indicate elevated, medium, and decreased expressions, accordingly. (C) OR2A1‐AS1 was increased in the DLBCL cell relative to CD19‐positive B cell. (D) Box plots showed normalized intensities from DLBCL cell and CD19‐positive B cell

In the current study, CD19‐positive B cells and DLBCL cells, i.e.*,* OCI‐ly1 and OCI‐ly19 were designated to examined the lncRNAs expression level using GEO data microarray profiling. According to the Volcano curve, the expression of lncRNA between DLBCL cells and CD19‐positive B cells has some differences, as shown in Figure 1A. In addition, the lncRNAs differential expression may significantly distinguish DLBCL cells (2.335) from CD19‐positive B cells (11.87). The unsupervised hierarchical clustering analysis of differentially expressed lncRNAs revealed that the dysregulated lncRNAs expression patterns were distinguishable between the DLBCL cell and CD19‐positive B cell, and ASHGA5P019110 (OR2A1‐AS1) were mostly decreased in DLBCL cell compared with CD19‐positive B cell (Figure 1B,C). In addition, Box plots showed normalized intensities from DLBCL cell and CD19‐positive B cell (Figure 1D). Thus, we chose OR2A1‐AS1 for further research.

### 3.2 Decreased expression of OR2A1‐AS1 in DLBCL patients

**FIGURE 2:**
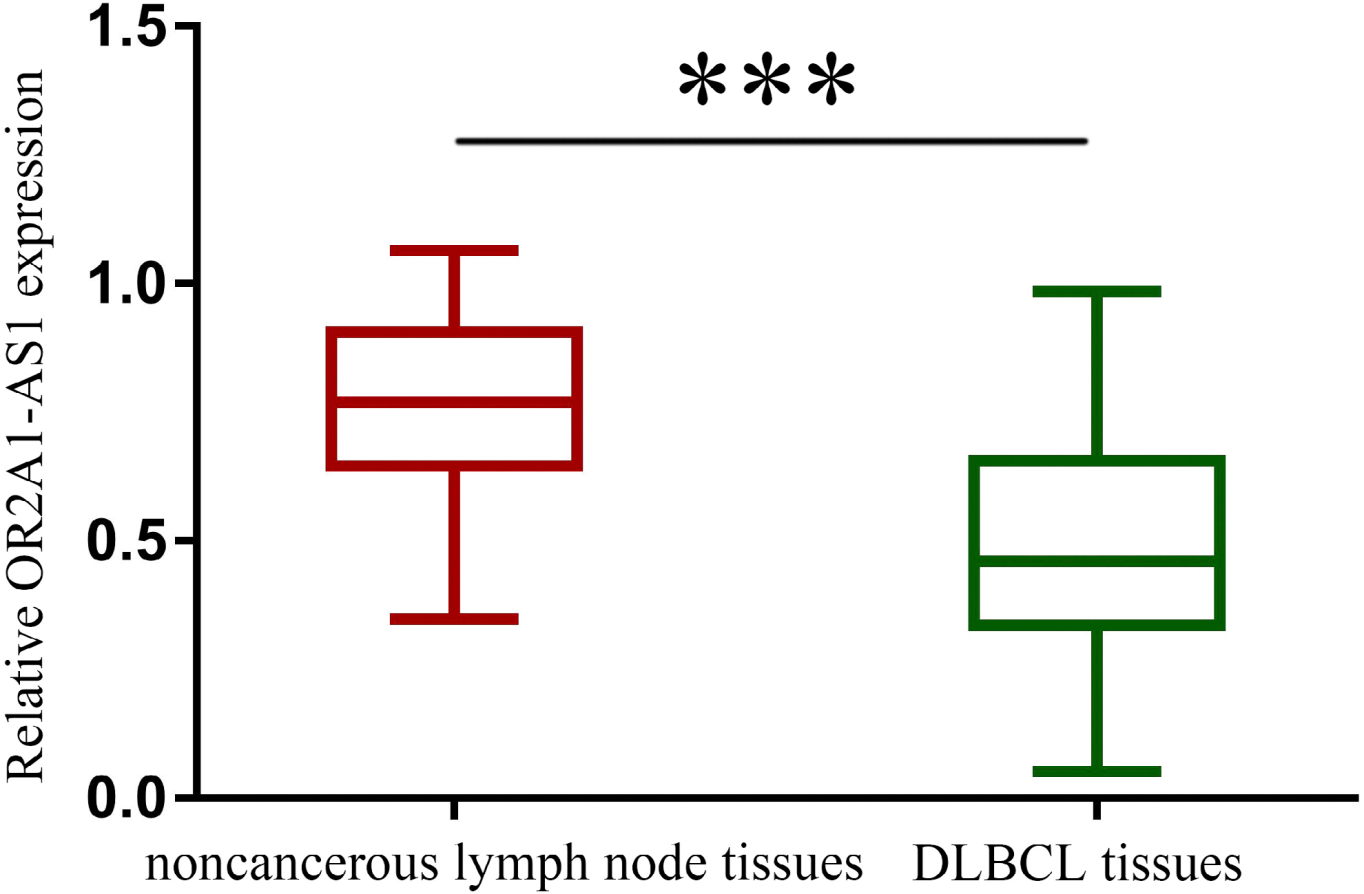
Decreased expression of OR2A1‐AS1 in DLBCL patients. ***p < 0.001

Considering 98 noncancerous lymph node tissues and 98 primary DLBCL tissues, RT‐qPCR was conducted to evaluate the OR2A1‐AS1 selected via Hiseq sequencing. The result showed that OR2A1‐AS1 expression was considerably reduced 2.9 times in DLBCL tissues (Figure 2). Because of this, the existing study aimed to evaluate the clinical and experimental application of OR2A1‐AS1 in DLBCL.

### 3.3 Patient characteristics

Table 1 shows the clinicopathological features. Herein, qRT‐PCR was run to evaluate the OR2A1‐AS1 expression in the RCHOP‐like cohort, which included non‐GCB‐like‐DLBCL (109/256, 42.6%) and GCB‐DLBCL (147/256, 57.4%). The OR2A1‐AS1 index was found in non‐GCB‐like‐DLBCL and GCB‐DLBCL (Table 1).

**TABLE 1:** Clinical features of DLBCL patients

Variables	Number of cases (%)
Age ≥ 60 y	45 (45.7)
Male	58 (59.4)
Stage III‐IV	51 (51.6)
Abnormal LDH level	45 (46.1)
Performance state 3–4	44 (45.3)
Extranodal involvement ≥2	20 (20.7)
B symptom	42 (42.6)
IPI 3–5	46 (47.3)
GCB	56 (57.4)
Non‐GCB	42 (42.6)

Abbreviations: GCB, germinal center B‐cell‐like subtype; IPI, International Prognostic Index; LDH, lactate dehydrogenase.

### 3.4 Prognosis of OR2A1‐AS1

**FIGURE 3:**
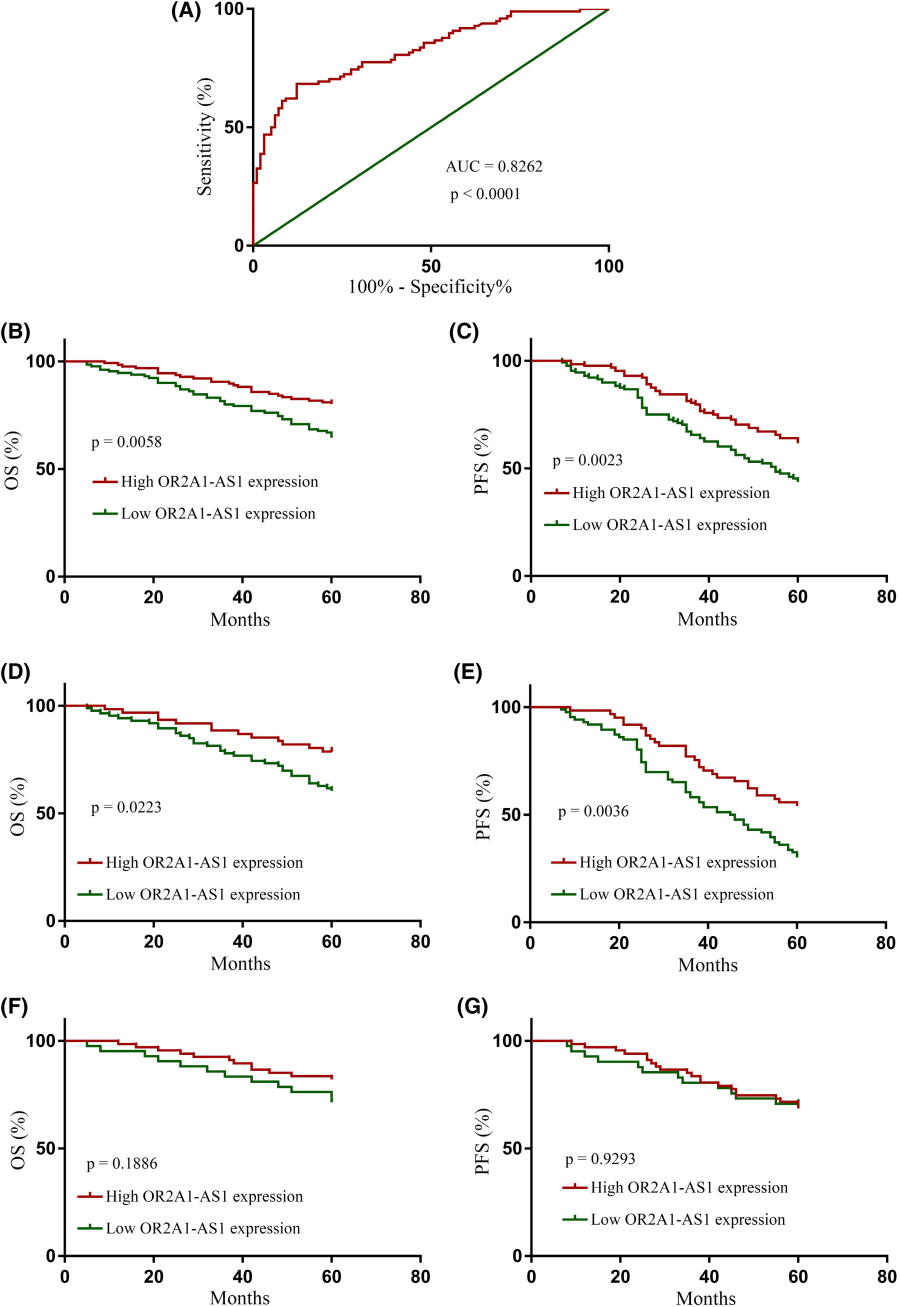
(A) ROC analysis for the expression OR2A1‐AS1 in DLBCL patients. (B) OS and (C) PFS with elevated and decreased OR2A1‐AS1 index patients in the total cohort. (D) OS and (E) PFS with elevated and decreased OR2A1‐AS1 index patients in GCB‐DLBCL cohort. (F) OS and (G) PFS with elevated and decreased OR2A1‐AS1 index patients in the non‐GCB‐like‐DLBCL cohort

We explored the correlation between clinicopathological features and OR2A1‐AS1 expression after validating OR2A1‐AS1 overexpression. OR2A1‐AS1 expression was substantially linked with CHOP‐like treatment, B‐symptoms, stages, subtypes, performance status, and IPI in 256 samples (Table 2) but not with other clinicopathological characteristics, i.e., sex and age. The ROC graphical curve was plotted to determine the OR2A1‐AS1 activity in DLBCL patients and normal control. A cutoff value was calculated. The diagnostic specificity, sensitivity, and area under the curve (AUC) values were found to be the same, i.e.*,* 0.9494 (Figure 3A). Consequently, a cutoff value of = 0.45 was selected for OR2A1‐AS1 overexpression. Those with lower OR2A1‐AS1 expression have a substantially shorter OS and PFS (*p* = 0.0058 and 0.0023, accordingly) when matched to patients with an elevated OR2A1‐AS1 expression (Figure 3B,C). Next, the OR2A1‐AS1 predictive value was examined via various COOC. In the GCB group, low OR2A1‐AS1 expression suggested a poor outcome than elevated OR2A1‐AS1 expression (OS: *p* = 0.0223; PFS: *p* = 0.0036) (Figure 3D,E). Furthermore, in the non‐GCB group, patients with lower OR2A1‐AS1 expression had the same OS and PFS (*p* = 0.1886, and 0.9293, respectively) as those with an elevated OR2A1‐AS1 expression (Figure 3F,G). In multivariate analysis, the Cox proportional hazards regression (CPHR) revealed that lower OR2A1‐AS1 expression was an independent PFS prognostic predictor (*p* = 0.001) (Tables 3 and 4).

**TABLE 2:** Association of OR2A1‐AS1 expression with clinical parameters in DLBCL patients

Variable	Number	High OR2A1‐AS1 expression (%)	Low OR2A1‐AS1 expression (%)	*p* value
Sex				0.842
Male	58	27	31	
Female	40	22	18	
Age				0.746
≥60 years	45	23	22	
<60 years	53	26	27	
B symptoms				<0.05
Present	42	13	29	
Absent	56	36	20	
Stage				<0.05
I‐II	47	35	12	
III‐IV	51	14	37	
Performance status				<0.05
0–2	54	37	17	
3–4	44	12	32	
CHOP‐like treatment				<0.05
Response	44	34	10	
Nonresponse	54	15	39	
Subtypes				<0.05
GCB	56	16	40	
Non‐GCB	42	33	9	
IPI				<0.05
0–3	52	37	15	
3–5	46	12	34	

*Note*: A statistically considerable variation was defined as *p* < 0.05.

Abbreviations: CHOP, cyclophosphamide, rituximab, vincristine, doxorubicin, and prednisone; GCB, germinal center B‐cell‐like subtype; IPI, International Prognostic Index.

**TABLE 3:** Univariate and multivariate Cox analyses of factors related to DLBCL patient OS

	Univariate analysis			Multivariate analysis		
Variables	HR	95% CI	*p* value	HR	95% CI	*p* value
Extranodal involvement ≥2	2.196	1.546–5.021	0.0002	0.457	0.133–1.259	0.241
Elevated LDH level	1.986	1.235–3.654	0.0004	0.846	0.254–2.156	0.654
Stage III‐IV	1.534	0.976–2.345	0.0262	0.653	0.245–2.152	0.224
IPI ≥3	2.954	2.653–5.451	0.0352	1.523	0.216–5.143	0.028
B symptom	1.432	1.025–3.654	0.0215	0.674	0.048–0.952	0.125
Performance state 3–4	1.825	1.025–2.657	0.0046	0.492	0.145–1.124	0.395
Low OR2A1‐AS1 expression	2.493	1.023–5.021	0.0032	2.546	1.254–4.653	0.005

*Note*: A statistically considerable variation was defined as *p* < 0.05.

Abbreviations: IPI, International Prognostic Index; LDH, lactate dehydrogenase.

**TABLE 4:** Univariate and multivariate Cox analyses of factors related to DLBCL patient PFS

	Univariate analysis			Multivariate analysis		
Variables	HR	95% CI	*p* value	HR	95% CI	*p* value
Extranodal involvement ≥2	1.952	1.325–4.109	0.0011	0.546	0.328–1.219	0.207
Elevated LDH level	1.765	1.025–3.562	0.0006	0.523	0.244–0.958	0.349
Stage III‐IV	1.452	0.843–3.064	0.0149	1.532	0.794–2.025	0.029
IPI ≥3	2.217	1.246–6.251	0.0115	1.985	0.773–3.195	0.011
B symptom	1.232	0.946–3.452	0.0348		0.586–1.547	0.637
Performance state 3–4	1.563	0.925–3.846	0.0049	1.848	0.749–2.065	0.003
Low OR2A1‐AS1 expression	2.846	1.125–9.325	0.0017	3.251	1.012–5.065	0.001

*Note*: A statistically considerable variation was defined as *p* < 0.05.

Abbreviations: IPI, International Prognostic Index; LDH, lactate dehydrogenase.

## 4 DISCUSSION

The reported studies have revealed the role of LncRNAs in many carcinomas.^20^, ^21^ LncRNAs have been evaluated as potential biomarkers and therapeutic targets for many cancers, such as DLBCL.^22^, ^23^, ^24^ Herein, we identified lncRNAs that could enhance the diagnostic and prognostic potencies in DLBCL patients. The identified lncRNAs were then determined and confirmed in a variety of specimens, including primary tissues from DLBCL patients, ensuring a high level of accuracy. ROC curves revealed that OR2A1‐AS1 was the candidate lncRNA, with high AUC, diagnostic sensitivity, and specificity.

The abnormal expression of specific lncRNAs may indicate the progression of cancer and can serve as key diagnostic and prognostic biomarkers.^25^, ^26^ Zhou et al. indicated some lncRNAs that might have a considerable role in the diagnosis and prediction of DLBCL.^27^ However, it is currently unclear whether other lncRNAs can be used as candidate biomarkers in DLBCL. Hence, it is needed to identify novel biomarkers involved in the DLBCL development. One lncRNA can be expressed at varying amounts in different samples and disorders. In the current study, a large sample group was enrolled to evaluate the expression of potential lncRNAs that led to the identification of one considerably varied lncRNA, i.e.*,* OR2A1‐AS1.

LncRNA including XLOC_009167, D16366, and PTCSC3 are potential biomarkers that predict lung cancer,^28^ hepatocellular cancer,^29^ and gastric cancer,^30^ accordingly. However, the prognostic value of the OR2A1‐AS index has barely been identified in DLBCL. In this study, it has been indicated that the group having the low expression of OR2A1‐AS had considerably worse outcomes than the group having an elevated expression of OR2A1‐AS. Stratification analysis revealed the prognostic value of OR2A1‐AS in GCB‐DLBCL but not in non‐GCB‐like‐DLBCL. OR2A1‐AS remained a significant predictive factor of PFS (in DLBCL) in MVA by CPHR. The limitation of this work is that the number of samples is not large enough. In addition, the exact molecular mechanism of OR2A1‐AS in GCB‐DLBCL needs to be explored in future research.

## 5 CONCLUSION

The OR2A1‐AS index was found to be an effective predictor of patients' outcomes with DLBCL, particularly in the GCB‐DLBCL group. OR2A1‐AS was found to be a significant predictor of PFS in DLBCL in MVA. Targeting OR2A1‐AS treatments could be a promising method to improve patient’s outcomes in the age of precision medicine.

## CONFLICT OF INTEREST

No competing interests have been declared by the authors.

## Data Availability

Due to the nature of this research, participants of this study did not agree for their data to be shared publicly, so supporting data are not available.
